# Prevalence of gestational diabetes mellitus in women with a family history of type 2 diabetes in first- and second-degree relatives

**DOI:** 10.1007/s00592-022-02011-w

**Published:** 2022-12-12

**Authors:** Cécile Monod, Grammata Kotzaeridi, Tina Linder, Daniel Eppel, Ingo Rosicky, Valeria Filippi, Andrea Tura, Irene Hösli, Christian S. Göbl

**Affiliations:** 1 Department of Obstetrics and Gynaecology, University Hospital Basel, Basel, Switzerland; 2 Department of Obstetrics and Gynaecology, Medical University of Vienna, Waehringer Guertel 18-20, 1090 Vienna, Austria; 3 Metabolic Unit, CNR Institute of Neuroscience, Padua, Italy

**Keywords:** Gestational diabetes, Type 2 diabetes mellitus, Family history, Degree of kinship

## Abstract

**Aims:**

A family history of type 2 diabetes mellitus (T2DM) markedly increases an individual's lifetime risk of developing the disease. For gestational diabetes (GDM), this risk factor is less well characterized. This study aimed to investigate the relationship between family history of T2DM in first- and second-degree relatives in women with GDM and the differences in metabolic characteristics at early gestation.

**Methods:**

This prospective cohort study included 1129 pregnant women. A broad risk evaluation was performed before 16 + 0 weeks of gestation, including a detailed family history of the different types of diabetes and a laboratory examination of glucometabolic parameters. Participants were followed up until delivery and GDM assessed according to the latest diagnosis criteria.

**Results:**

We showed that pregnant women with first- (FHD1, 26.6%, OR 1.91, 95%CI 1.16 to 3.16, *p* = 0.005), second- (FHD2, 26.3%, OR 1.88, 95%CI 1.16 to 3.05, *p* = 0.005) or both first- and second-degree relatives with T2DM (FHD1 + D2, 33.3%, OR 2.64, 95%CI 1.41 to 4.94, *p* < 0.001) had a markedly increased risk of GDM compared to those with negative family history (FHN) (*n* = 100, 15.9%). The association was strongest if both parents were affected (OR 4.69, 95%CI 1.33 to 16.55, *p* = 0.009). Women with FHD1 and FHD1 + D2 had adverse glucometabolic profiles already in early pregnancy.

**Conclusions:**

Family history of T2DM is an important risk factor for GDM, also by applying the current diagnostic criteria. Furthermore, we showed that the degree of kinship plays an essential role in quantifying the risk already at early pregnancy.

**Supplementary Information:**

The online version contains supplementary material available at 10.1007/s00592-022-02011-w.

## Introduction

In parallel with the increase in obesity and type 2 diabetes (T2DM) in the general population, the incidence of gestational diabetes (GDM) is rising. Currently, it is estimated that GDM affects up to 17% of pregnant women [1] and is responsible for major maternal and foetal complications during pregnancy and at birth as well as for long-term complications such as cardiovascular diseases and T2DM in both mothers and offspring [1].

The pathophysiology of GDM appears to be largely similar to that of T2DM, as it develops as a result of both impaired insulin sensitivity and β-cell dysfunction, caused by the metabolic stress of pregnancy [2]. In T2DM, a family history of diabetes markedly increases an individual's lifetime risk for developing diabetes, especially if the mother or both parents are affected. This may be caused by the interaction of environmental as well as genetic factors [3–5]. Thereby, genetic research discovered more than 100 genes that increase susceptibility to T2DM. Despite this polygenetic nature of T2DM, genetics explains only about one tenth of familial cases [3, 6, 7]. Aasberg et al. showed that not only affected parents but also an affected spouse is a risk factor for T2DM, reflecting the complex interrelationship between genetic and environmental factors [3].

Among the risk factors for GDM, a family history of T2DM is therefore of possible importance, as it may include both genetic and environmental factors [8, 9]. There are data indicating that pregnant women with a family history of T2DM have an increased risk of giving birth to a large for gestational age (LGA) neonate or have an increased risk for caesarean section, which are commonly known complications of hyperglycaemia in pregnancy as well [8]. However, available studies about family history as a risk factor for GDM provided rather heterogeneous results as screening and diagnostic criteria for GDM, degree of kinship and type of diabetes markedly differ between studies [9–12].

To get a better understanding of the factors determining the development of GDM, this study aims to investigate the relationship between family history of T2DM as well as the degree of kinship with the development of GDM, according to the most actual diagnosis criteria. We also aim to assess the differences in metabolic characteristics at early gestation in women with and without a family history of T2DM.

## Methods

The details of the study design are reported elsewhere [13]. In short, this prospective cohort study included a total of 1164 participants among all women attending our pregnancy outpatient clinic (Department of Obstetrics and Gynaecology, Medical University of Vienna), between 2016 and 2019. Women with preconceptionally unrecognized diabetes (diagnosed by HbA1c ≥ 6.5% (48 mmol/mol) and/or fasting plasma glucose ≥ 126 mg/dL at early pregnancy) or unknown family history or GDM status were excluded, resulting in an effective sample size of 1129 women. A broad risk evaluation was performed at 12.9 weeks, interquartile range (IQR) (12.3 to 13.6 weeks), including the assessment of maternal characteristics (age, parity, obstetric history, history of GDM, ethnicity, as well as pregestational and current body mass index (BMI)). We collected a detailed family history of the different types of diabetes and especially of T2DM. First-degree relatives were defined as parents or siblings of the pregnant woman, and second-degree relatives were defined as grandparents, aunts and uncles [14]. Pregnant women without family history of T2DM were classified as FHN. Pregnant women with family history of T2DM were further categorized as follows: FHD1 with one or more first-degree relatives, FHD2 with one or more second-degree relatives, FHD1 + D2 with both first- and second-degree relatives, FHD1-F with father, FHD1-M with mother and FHD1-F + M with both parents affected. A blood examination was performed at the baseline visit to assess fasting plasma glucose (FPG), insulin and C-peptide, lipids and glycated haemoglobin A1c (HbA1c). Fasting measurements were further used to calculate the homeostasis model assessment of insulin resistance (HOMA-IR) and the quantitative insulin sensitivity index from C-peptide (QUICKIc) [15, 16]. The study participants received universal GDM testing by use of a 75-g 2-h OGTT at the late second or early third trimester. Thereby, GDM was diagnosed according to the actual WHO (World Health Organization) recommendations if fasting and/or glucose concentrations after oral glucose load exceeded the proposed cut-offs [17]. All laboratory parameters, which were assessed at study entry, were measured according to the standard laboratory methods at our certified Department of Medical and Chemical Laboratory Diagnostics (http://www.kimcl.at). Glucose measurements during the diagnostic OGTT were assessed at local public laboratories by the use of venous plasma blood samples according to international and local guidelines [17, 18]. The study was approved by the Ethics Committee of the Medical University of Vienna and performed in accordance with the Declaration of Helsinki. Written informed consent was obtained from all participants.

### Statistical analysis

Continuous variables were summarized by mean ± standard deviation or as median and IQR (in case of skewed distribution). These were compared by analysis of variance or rank-based inference. Categorical variables were summarized by counts and percentages, and compared by binomial logistic regression. Odds ratios and 95% confidence intervals (95%CI) were additionally calculated for binary outcomes. Tukey’s honestly significant difference (HSD) test was used for all subgroup (k = 4) comparisons to achieve a 95% coverage probability. Statistical analysis was performed with R (version 4.0.2) and contributing packages (especially “multcomp” and “nparcomp”). A two-sided p-value of ≤ 0.05 was considered statistically significant.

## Results

### Glucose metabolism and GDM prevalence associated with the degree of kinship

**Fig. 1:**
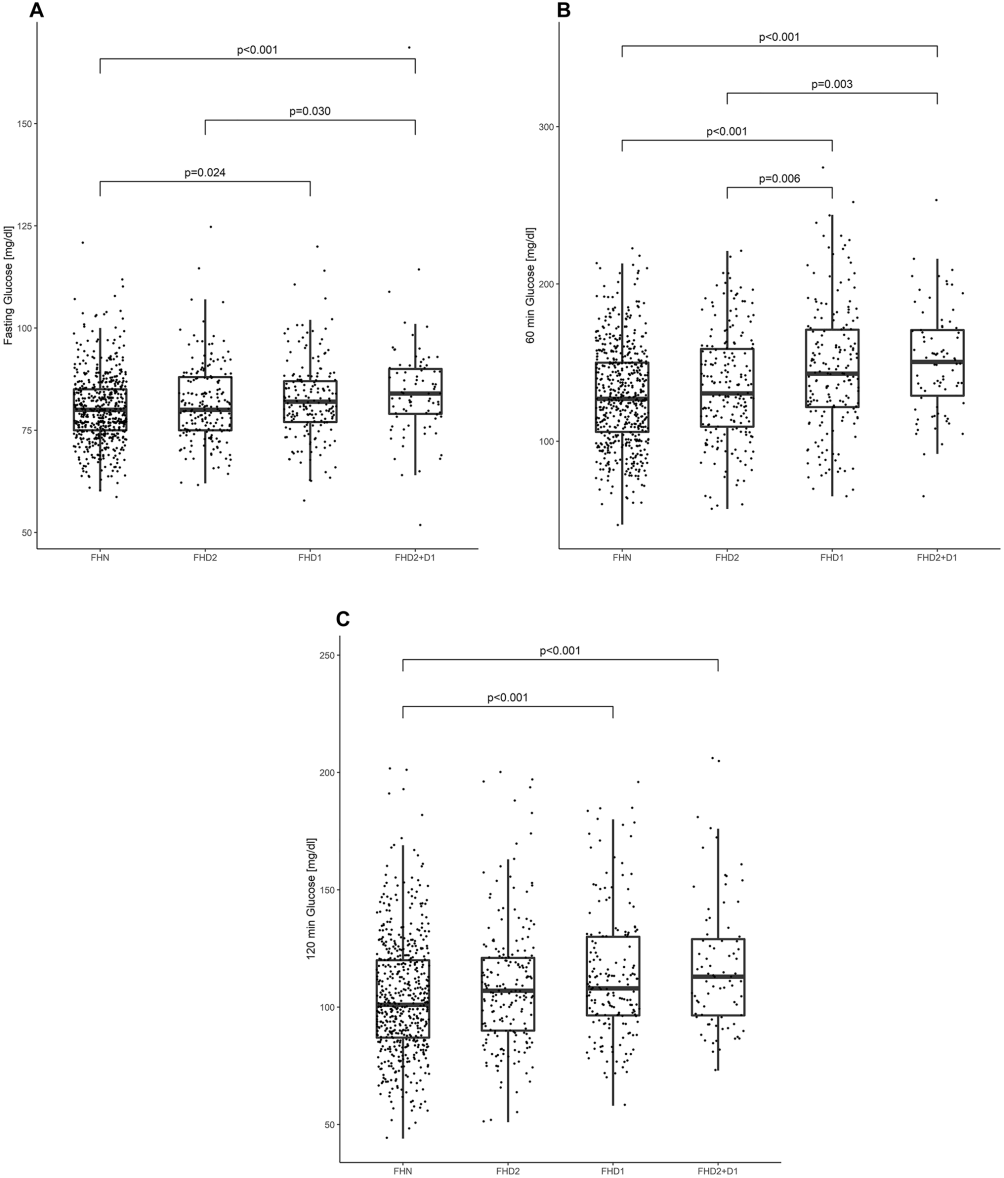
Glucose levels during the OGTT in women without family history of type 2 diabetes (FHN), women with only second- (FHD1) and only first-degree relatives (FHD1) or with both (FHD1 + D2)

Characteristics of the study sample categorized according to the degree of kinship are provided in Table 1. Pregnant women with first-degree relatives with T2DM (FHD1) or both first- and second-degree relatives with T2DM (FHD1 + D2) showed markedly adverse glucometabolic profile as compared to women without family history of type 2 diabetes (FHN), whereas this was not observed for women with only second-degree relatives (FHD2). In particular, FHD1 + D2 and FHD1 presented higher triglycerides (FHD1 + D2: *p* = 0.024, FHD1: *p* = 0.002), HbA1c (FHD1 + D2: *p* = 0.012, FHD1: *p* = 0.040), insulin (FHD1 + D2: *p* < 0.001, FHD1: *p *= 0.005) and C-peptide (FHD1 + D2: *p* = 0.002, FHD1: *p* < 0.001), as well as a higher pregestational BMI (FHD1 + D2: *p* < 0.001, FHD1: *p* < 0.001), and were more insulin resistant as compared to the FHN group. Moreover, FHD1 women showed increased in total and LDL cholesterol as compared with FHN and FHD2 mothers, while the group with both first- and second-degree relatives with T2DM (FHD1 + D2) presented significantly higher HbA1c as compared to the FHD2 group. As visualized in Fig. 1, glucose levels during the diagnostic OGTT at later pregnancy were notably increased in FHD1 and FHD2 + D1 vs. FHN, and (although not significant) a tendency for higher glucose levels at 2 h after oral glucose ingestion was observed for FHD2 vs. FHN (*p* = 0.074). The prevalence of GDM was 26.6% (*n* = 51, OR 1.91, 95%CI 1.16–3.16, *p* = 0.005) in the FHD1, 26.3% (*n* = 57, OR 1.88, 95%CI 1.16–3.05, *p* = 0.005) in the FHD2 and 33.3% (*n* = 31, OR 2.64, 95%CI 1.41–4.94, *p* < 0.001) in the FHD1 + D2 group. All those prevalence rates were significantly elevated as compared to women without family history of diabetes, FHN (*n* = 100, 15.9%). The results remained comparable after adjustment for pregestational BMI and maternal age. There were no differences among the groups in obstetric outcome or offspring biometry (supplemental material, Table S1).

**Table 1:** Characteristics of subgroups categorized according to the degree of kinship

	FHN	FHD2	FHD1	FHD2 + D1
	(*n* = 627)	(*n* = 217)	(*n* = 192)	(*n* = 93)
Age (years)	31.7 ± 5.9	30.7 ± 5.7	32.8 ± 5.5^2^	32.2 ± 5.6
Parity (≥ 1)	385 (61.4)	117 (53.9)	139 (72.4)^1,2^	64 (68.8)
GDM in previous pregnancy	52 (8.3)	17 (7.8)	32 (16.7)^1,2^	19 (20.4)^1,2^
Ethnicity (non-Caucasian)	158 (25.2)	16 (7.4)^1^	52 (27.1)^2^	24 (25.8)^2^
BMI, before pregnancy (kg/m^2^)	24.3 ± 5.0	24.8 ± 6.1	26.0 ± 5.6^1^	26.7 ± 5.4^1,2^
Multiple pregnancy	83 (13.2)	22 (10.1)	14 (7.3)	8 (8.6)
Triglycerides, early pregnancy (mg/dl)	115 ± 50	117 ± 44	129 ± 48^1^	129 ± 46^1^
Total cholesterol, early pregnancy (mg/dl)	187 ± 35	185 ± 33	196 ± 35^1,2^	193 ± 35
LDL cholesterol, early pregnancy (mg/dl)	93 ± 28	92 ± 26	101 ± 27^1,2^	101 ± 31
HDL cholesterol, early pregnancy (mg/dl)	71 ± 17	70 ± 15	69 ± 16	67 ± 13
FPG, early pregnancy (mg/dl)	81 ± 6.2	82 ± 6.6	83 ± 7.0^1^	82 ± 8.1
HbA1c, early pregnancy (%)	4.97 ± 0.31	4.98 ± 0.30	5.04 ± 0.27^1^	5.08 ± 0.35^1,2^
HbA1c, early pregnancy (mmol/mol)	30.8 ± 3.3	30.9 ± 3.3	31.6 ± 3.0^1^	32.0 ± 3.9^1,2^
Fasting insulin, early pregnancy (µU/ml)	7.5 (5.4–10.7)	8.3 (5.3–11.7)	9.3 (5.9–13.7)^1^	10.2 (6.7–16.5)^1,2^
Fasting C-peptide, early pregnancy (ng/ml)	1.5 (1.2–1.9)	1.6 (1.3–2.1)	1.8 (1.4–2.3)^1^	1.9 (1.4–2.5)^1^
HOMA-IR, early pregnancy (dimensionless)	1.5 (1.0–2.2)	1.7 (1.0–2.4)	2.0 (1.2–2.9)^1^	2.0 (1.3–3.5)^1,2^
QUICKIc, early pregnancy (dimensionless) × 10^2^	48 (45–50)	47 (45–50)	46 (44–49)^1^	46 (43–49)^1^
OGTT glucose 0 min (mg/dl)	80 ± 8.6	81 ± 9.3	83 ± 9.6^1^	85 ± 13.5^1,2^
OGTT glucose 60 min (mg/dl)	129 ± 31.5	135 ± 34.9	146 ± 40.5^1,2^	151 ± 33.3^1,2^
OGTT glucose 120 min (mg/dl)	104 ± 24.9	110 ± 27.1	113 ± 27.1^1^	118 ± 28.2^1^
GDM in actual pregnancy	100 (15.9)	57 (26.3)^1^	51 (26.6)^1^	31 (33.3)^1^

Data are mean ± SD or median (IQR) and count (%) for women with negative family history of type 2 diabetes (FHN), who had a second- (FHD2) and first-degree relative with type 2 diabetes (FHD1) or both (FHD2 + D1). BMI, body mass index; FPG, fasting plasma glucose; HbA1c, glycated haemoglobin A1c; quantitative insulin sensitivity check index from C-peptide (QUICKIc); oral glucose tolerance test (OGTT)

^1^*p* < 0.05 versus FHN

^2^*p* < 0.05 versus FHD2

^3^*p* < 0.05 versus FHD1

### Glucose metabolism and GDM prevalence associated with the family history of T2DM in parents

For this analysis, patients with second-degree relatives or those with affected siblings but without affected parents were excluded. A total of 86 women had a father with T2DM (FHD1-F), 76 women had a mother with T2DM (FHD1-M), and in 17 women, both parents had T2DM (FHD1-F + M). The characteristics of these groups are provided in Table 2. As compared to FHN, the FHD1-M group presented higher triglycerides and fasting glucose and was more insulin resistant at the beginning of pregnancy. Moreover, they showed elevated glucose levels at 60 min during the diagnostic OGTT at later pregnancy. Higher glucose values during the OGTT were also observed in the FHD1-F + M group, who also presented increased HbA1c levels at beginning of pregnancy. This was associated with an increased risk of GDM in these women (OR 4.69, 95%CI 1.33–16.55, *p* = 0.009 vs. FHN). There were no differences in perinatal outcome or offspring biometry (supplemental material, Table S2).

**Table 2:** Characteristics of subgroups categorized according to parental family history of type 2

	FHN	FHD1-F	FHD1-M	FHD1-F + M
	(*n* = 627)	(*n* = 86)	(*n* = 76)	(*n* = 17)
Age (years)	31.7 ± 5.9	31.6 ± 5.5	33.9 ± 5.8^1,2^	33.3 ± 4.3
Parity (≥ 1)	385 (61.4)	63 (73.2)	53 (69.7)	14 (82.4)
GDM in previous pregnancy	52 (8.3)	14 (16.3)	11 (14.5)	4 (23.5)
Ethnicity (non-Caucasian)	158 (25.2)	21 (24.4)	19 (25.0)	8 (47.1)
BMI, before pregnancy (kg/m^2^)	24.3 ± 5.0	26.1 ± 6.1^1^	25.6 ± 5.3	26.9 ± 4.9
Multiple pregnancy	83 (13.2)	7 (8.1)	5 (6.6)	1 (5.9)
Triglycerides, early pregnancy (mg/dl)	114 ± 50	127 ± 51	130 ± 46^1^	128 ± 42
Total cholesterol, early pregnancy (mg/dl)	187 ± 35	196 ± 36	197 ± 33	192 ± 41
LDL cholesterol, early pregnancy (mg/dl)	93 ± 28	100 ± 25	101 ± 27	100 ± 32
HDL cholesterol, early pregnancy (mg/dl)	71 ± 17	71 ± 18	70 ± 15	67 ± 14
FPG, early pregnancy (mg/dl)	81 ± 6.2	83 ± 6.6	82 ± 6.9^1^	86 ± 8.1
HbA1c, early pregnancy (%)	4.97 ± 0.31	5.01 ± 0.27	5.02 ± 0.26	5.24 ± 0.30^1,2,3^
HbA1c, early pregnancy (mmol/mol)	30.8 ± 3.3	31.2 ± 2.97	31.3 ± 2.85	33.7 ± 3.23^1,2,3^
Fasting insulin, early pregnancy (µU/ml)	7.5 (5.4 − 10.7)	8.1 (5.3 − 12.1)	10.1 (6.5 − 12.9)^1^	8.9 (6.5 − 16.5)
Fasting C-peptide, early pregnancy (ng/ml)	1.5 (1.2 − 1.9)	1.6 (1.3 − 2.1)	1.8 (1.4–2.3)^1^	1.9 (1.4 − 2.5)
HOMA-IR, early pregnancy (dimensionless)	1.5 (1.0 − 2.2)	1.6 (1.0 − 2.7)	2.1 (1.4 − 2.7)^1^	2.1 (1.3 − 3.6)
QUICKIc, early pregnancy (dimensionless) × 10^2^	48 (45 − 50)	47 (45 − 50)	46 (44 − 49)^1^	46 (43 − 48)
OGTT glucose 0 min (mg/dl)	80 ± 8.6	82 ± 8.9	82 ± 9.2	86 ± 11.6
OGTT glucose 60 min (mg/dl)	129 ± 31.5	138 ± 36.9	151 ± 42.3^1^	152 ± 43.2^1^
OGTT glucose 120 min (mg/dl)	104 ± 24.9	112 ± 25.8	114 ± 28.4^1^	110 ± 24.8
GDM actual pregnancy	100 (15.9)	20 (23.3)	17 (22.4)	8 (47.1)^1^

Data are mean ± SD or median (IQR) and count (%) for women with negative family history of type 2 diabetes (FHN), who had a father (FHD1-F), mother (FHD1-M) or both parents with type 2 diabetes (FHD1-F + M). BMI, body mass index; FPG, fasting plasma glucose; HbA1c, glycated haemoglobin A1c; quantitative insulin sensitivity check index from C-peptide (QUICKIc); oral glucose tolerance test (OGTT)

^1^*p* < 0.05 versus FHN

^2^*p* < 0.05 versus FHD1-F

^3^*p* < 0.05 versus FHD1-M

## Discussion

This study aimed to characterize the association between GDM and family history of T2DM in first- and second-degree relatives with T2DM by applying the current WHO recommendations, as well as to assess the glycometabolic profiles of women with and without family history of diabetes. We showed that pregnant women with first-, second- or both first- and second-degree relatives had an increased risk of GDM compared to those with negative family history. Women with first-degree relatives and both first- and second-degree relatives with history of T2DM had adverse glucometabolic profiles as compared to those without family history, including impaired insulin sensitivity, as well as higher glucose concentrations during the diagnostic OGTT.

Since many years, authors recognized family history of diabetes as a potential risk factor for GDM. However, most of the available studies were conducted before the actual diagnostic recommendations for GDM were published [9–11, 19–21]. Since this time, the prevalence of GDM changed [22, 23] due to new screening and diagnostic strategies as well as a rising rate of adiposity [1]. These changes potentially modified the importance of family history of diabetes as a risk factor as well. Furthermore, some authors considered family history of type 1 or type 2 diabetes indifferently [20, 24] or did not take the degree of kinship into account [9]. Solomon et al. as well as Williams et al. identified family history of T2DM in first-degree relatives as a risk factor for GDM. The risk was the highest if both parents were affected, which is in line with our results [10, 21]. Interestingly, women with only first as well as those with first- and second-degree relatives showed elevated triglycerides and HbA1c concentrations and were more insulin resistant already at start of gestation corresponding with a higher BMI as compared to women with negative family history. These results are of importance as to the best our knowledge, to date, no other study came to the conclusion about insulin resistance in early pregnancy in relation to the degree of kinship. Thereby, both genetic and environmental factors may be responsible for the adverse glucometabolic profiles observed in these women.

Traditionally, the risk of T2DM was considered higher in the case of a family history of diabetes on maternal side [4, 25, 26]. A recent Danish registry study also showed an association between family history of T2DM in the mother and the development of T2DM, although this was stronger when both parents were affected [3]. Nonetheless, the association of family history with T2DM in mothers and the development of GDM is not well known and still a matter of debate. Some earlier studies found an association with diabetes in the mother [10, 11, 27], whereas other did not [28]. Recently, Lewandowska et al. have observed a stronger association of GDM with paternal history of diabetes in certain subgroups of women [12]. While women with maternal family history showed adverse glucometabolic profiles in our study, the prevalence of GDM was only increased in pregnant women when both parents were affected, suggesting a more complex relationship between parental T2DM and the development of GDM.

In the past, several risk assessment models were proposed aiming to predict the development of GDM at early pregnancy [29]. In a recent study, we assessed the accuracy of 15 GDM prediction scores based on readily available routine parameters in a group of pregnant women. Family history of diabetes was heterogeneously integrated in these prediction models, in terms of both type of diabetes and degree of kinship, but was considered a determining factor in 13 of the 15 risk assessment tools. For example, one prediction model with satisfactory accuracy included family history of diabetes in first-degree relatives, however, without specifying the type of diabetes [30]. In line with these results, we suggest that differentiated consideration of family history of T2DM stratified by degree of kinship may improve the performance of the available prediction algorithms.

It is worth noting that in a large retrospective study Levy et al. showed higher rates of LGA and caesarean section in women with a positive family history of diabetes, irrespective of the diagnosis of GDM [8]. This is in contrast to our findings, as we failed to identify an association between the degree of kinship and obstetric outcomes or offspring biometry in our study. A possible reason for this discrepancy is that our study was not powered for obstetrical and neonatal outcomes. However, our sample size was in range or even larger as compared to most available studies on family history and GDM [9, 11, 12, 20, 21]. The strengths of this study are its prospective design and the detailed family history stratified for the degree of kinship and specified for the type of diabetes. Additionally, it is one of the first studies assessing the role of family history in a collective of women screened with the latest diagnostic criteria. Furthermore, the results relevance goes beyond the differences in the prevalence of GDM among the different subgroups, as the glucometabolic parameters collected in the first trimester also allow conclusions regarding insulin resistance and lipid profiles. Such data are important to improve prediction strategies for GDM already at early pregnancy.

We conclude that family history of T2DM is an important risk factor for the development of GDM by applying the current diagnostic criteria. Furthermore, we showed that the degree of kinship plays an essential role in quantifying the risk. Especially, mothers with first or first- and second-degree relatives with diabetes had an unfavourable risk profile with higher BMI and impaired insulin sensitivity. This information is essential to provide an optimized risk classification already at start of gestation.

## Supplementary Information

Below is the link to the electronic supplementary material.

Supplementary file1 (DOCX 21 KB)

## Data Availability

Data are available on request from the authors.
