# Supplementary material for: Prevalence of gestational diabetes mellitus in women with a family history of type 2 diabetes in first- and second-degree relatives
Source: Acta Diabetol. 2022 Dec 12;60(3):345–51. doi: 10.1007/s00592-022-02011-w (PMC9931850; doi:10.1007/s00592-022-02011-w)
Supplement: Supplementary file 1 — Supplementary file1 (DOCX 21 KB) [file 592_2022_2011_MOESM1_ESM.docx]

**Journal: Acta Diabetologica**

**Prevalence Of Gestational Diabetes In Women With A Family History Of Type 2 Diabetes In First- And Second-Degree Relatives**

Cécile Monod (1, 2), Grammata Kotzaeridi (2), Tina Linder (2), Daniel Eppel (2), Ingo Rosicky (2), Valeria Filippi (1), Andrea Tura (3), Irene Hösli (1), Christian S. Göbl (2)

(1) Department of Obstetrics and Gynaecology, University Hospital Basel, Basel, Switzerland

(2) Department of Obstetrics and Gynaecology, Medical University of Vienna, Vienna, Austria

(3) Metabolic Unit, CNR Institute of Neuroscience, Padova, Italy

**Corresponding address:**

Christian S. Göbl, MD, PhD, Associate Professor of Obstetrics

Department of Obstetrics and Gynaecology

Medical University of Vienna

Waehringer Guertel 18-20

A-1090 Vienna, Austria

Tel.: 0043-1-40400-28220

Fax: 0043-1-40400-28620

Email: christian.goebl@meduniwien.ac.at

**Supplementary Materials**

**Obstetric outcome and offspring biometry for subgroups**

**Table S1** Obstetric outcome and offspring biometry for subgroups categorized according to the degree of kinship

Multiple pregnancies were excluded from this analysis. Data are mean ± SD or median (IQR) and count (%) for women with negative family history of type 2 diabetes (FHN), who had a second (FHG2) and first degree relative with type 2 diabetes (FHG1) or both (FHG2+G1).

|  | **FHN** | **FHG2** | **FHG1** | **FHG2+G1** |
| --- | --- | --- | --- | --- |
|  | **(n = 544)** | **(n = 195)** | **(n = 178)** | **(n = 85)** |
| Induction of fetal lung maturation | 34 (6.4) | 13 (6.8) | 15 (8.8) | 6 (7.1) |
| Cesarean section | 222 (41.8) | 83 (43.2) | 86 (50.3) | 41 (48.2) |
| Vacuum extraction | 17 (3.2) | 17 (8.9)^1^ | 8 (4.8) | 4 (4.8) |
| Neonatal intensive care unit admission | 28 (5.3) | 14 (7.3) | 8 (4.8) | 2 (2.4) |
| GAD (weeks) | 39 (38-40) | 39 (38-40) | 38 (38-40) | 38 (38-39) |
| Preterm delivery (< 37 weeks) | 41 (7.7) | 19 (9.8) | 17 (9.9) | 7 (8.3) |
| Birth weight (percentile) | 43.0 ± 27.1 | 44.2 ± 28.0 | 44.0 ± 26.9 | 49.5 ± 26.5 |
| Birth length (percentile) | 43.7 ± 26.6 | 44.2 ± 28.7 | 43.5 ± 27.9 | 42.6 ± 27.9 |
| LGA | 33 (6.3) | 11 (5.7) | 8 (4.8) | 4 (4.9) |

**Table S2** Obstetric outcome and offspring biometry for subgroups categorized according to parental family history of T2DM

Multiple pregnancies were excluded from this analysis. Data are mean ± SD or median (IQR) and count (%) for women with negative family history of type 2 diabetes (FHN), who had a father (FHG1-F), mother (FHG2-M) or both parents with type 2 diabetes (FHG1-V+M).

|  | **FHN** | **FH1G-V** | **FH1G-M** | **FH1G-V+M** |
| --- | --- | --- | --- | --- |
|  | **(n = 627)** | **(n = 86)** | **(n = 76)** | **(n = 17)** |
| Induction of fetal lung maturation | 34 (6.4) | 8 (10.8) | 5 (7.2) | 2 (13.3) |
| Cesarean section | 222 (41.8) | 29 (38.7) | 40 (58.0) | 10 (66.7) |
| Vacuum extraction | 17 (3.2) | 4 (5.5) | 3 (4.5) | 1 (6.7) |
| Neonatal intensive care unit admission | 28 (5.3) | 5 (6.8) | 2 (3.0) | 1 (6.7) |
| GAD (weeks) | 39 (38-40) | 39 (38-40) | 38 (38-40) | 38 (37-39) |
| Preterm delivery (< 37 weeks) | 41 (7.7) | 11 (14.5) | 3 (4.3) | 3 (20.0) |
| Birth weight (percentile) | 43.0 ± 27.1 | 43.7 ± 27.0 | 47.3 ± 27.8 | 29.2 ± 19.9 |
| Birth length (percentile) | 43.7 ± 26.6 | 44.5 ± 29.2 | 44.0 ± 27.1 | 34.4 ± 26.2 |
| LGA | 33 (6.3) | 3 (4.1) | 5 (7.2) | 0 (0.0) |
